# Ligand Control of Ultrafast Hot-Carrier Cooling in CdSe Quantum Dots by a Coherent Nonadiabatic Mechanism

**DOI:** 10.1021/acs.jpclett.5c03429

**Published:** 2026-01-12

**Authors:** Shanu A. Shameem, Nila Mohan T. M., Ryan W. Tilluck, Caitlin V. Hetherington, Benjamin G. Levine, Warren F. Beck

**Affiliations:** † Department of Chemistry, 3078 Michigan State University, 578 South Shaw Lane, East Lansing, Michigan 48824, United States; ‡ Institute for Advanced Computational Science and Department of Chemistry, 12301 Stony Brook University, Stony Brook, New York 11794, United States

## Abstract

Using the results from global modeling of the broadband two-dimensional electronic spectrum and an analysis of the concurrent damping of excited-state vibronic coherences, we show herein that a coherent nonadiabatic mechanism converts the electronic excitation energy of the X3 (1P_3/2_–1P_e_) exciton state to ligand vibrations on the <50 fs time scale in oleate- and hexadecylamine-capped CdSe quantum dots. A comparison of the rates for the two ligands suggests that this process is promoted by mid-frequency vibrations of the ligands due to modulation of their electron-donating tendency. An intramolecular vibrational redistribution process then follows on the ∼200 fs time scale with both ligands, which accompanies thermalization in the band-edge X1 (1S_3/2_–1S_e_) state and dephasing of the spectator ligand vibrations. These findings suggest that charge-separated intermediates associated with photoinduced charge transfer or triplet–triplet excitation energy transfer will be produced with retention of phase coherence in the vibrations that modulate the charge-transfer character of surface-bound organic acceptors.

## Graphical abstract

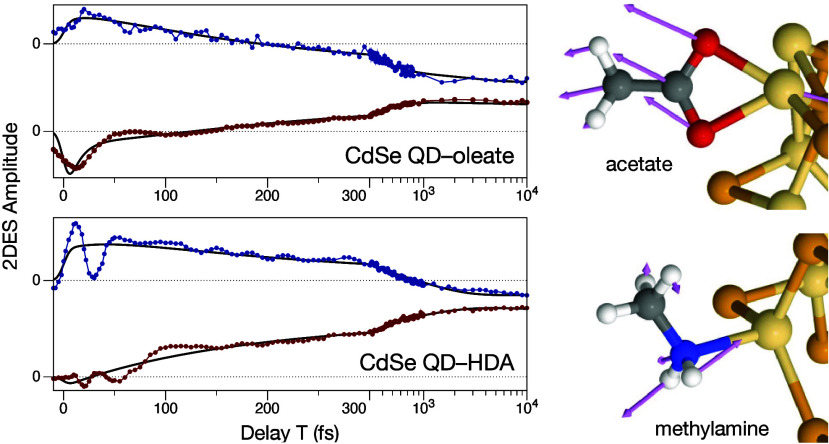

The intraband exciton internal conversion or hot-carrier cooling dynamics of semiconductor quantum dots (QDs)^1–8^ is accelerated into the subpicosecond time scale in the presence of organic surface-capping ligands.^9–15^ In recent work, we advanced a new picture in which the ligands can provide efficient channels for the dissipation of electronic excitation energy using their mid- to high-frequency vibrations to mediate nonadiabatic transitions between the exciton states. We showed that CdSe QDs prepared with hexadecylamine (HDA)^16^ or oleate ligands^17^ exhibit coherent excited-state wavepacket motions in ligand-specific vibrational modes during internal conversion to the band-edge and photoluminscence (PL) states. The results were interpreted in terms of a many-atom or molecular theory involving exciton potential surfaces. Calculations of a small Cd_33_Se_33_ nanocrystal showed that ligand displacements provide a pathway to the band-edge state along a favorable energy gradient through a cascade of conical intersections (CIs).^18,19^ The calculations account for the specific set of ligand vibrations that were observed experimentally to accompany hot-carrier cooling in terms of the energy that each mode accepts along the cooling reaction coordinate. Left indeterminate by the experiments and theory work, however, is the nature of the dynamic mechanism that allows the ligand vibrations to control the rate of hot-carrier cooling. A key observation to be explained is why the excited-state wavepacket motions of the oleate’s alkylcarboxylate group are much more rapidly damped than those of the alkylamine moiety of HDA.

In this contribution, we obtain definitive evidence for a coherent nonadiabatic mechanism for hot-carrier cooling from a comparison of the time evolution of the two-dimensional electronic spectra (2DES) and the damping of excited-state vibronic coherences in HDA- and oleate-capped QDs. Using a global model of the 2DES spectrum, we now find that the exciton internal conversion mechanism in the presence of surface-capping ligands involves two processes: an electronic process and an intramolecular vibrational redistribution (IVR) process. Conversion of the electronic excitation energy of the X3 (1P_3/2_–1P_e_) state to ligand vibrations occurs on the <50 fs time scale, but this process is as much as 3 times faster for the oleate ligands than for the HDA ligands in CdSe QDs. These results suggest the hypothesis that the electron-donating character of the ligands controls the driving force for hot-carrier cooling via an excited-state trajectory along a sequence of CIs between the exciton potential energy surfaces. This idea accounts for the activity of the rapidly damped ligand vibrations as the tuning modes in the nonadiabatic reaction dynamics. An IVR-mediated cooling process then follows on the ∼200 fs time scale with both ligands, which results in thermalization in the band-edge state and dephasing of the spectator ligand vibrations.

Figure 1 compares 2DES spectra selected from the data sets for the HDA-capped^16^ and oleate-capped^17^ CdSe QDs at a short probe delay time, *T* = 5 fs. The diagonals of both spectra show partially resolved peaks due to optical transitions to the lowest three exciton states, X1 (1S_3/2_–1S_e_), X2 (2S_3/2_–1S_e_), and X3, and that from the oleate-capped QDs shows an additional splitting of the diagonal peak at X1 apparently due to the band-edge fine structure.^20^ Owing to the use in these experiments of very short laser pulses, with durations much shorter than the inhomogeneous line broadening time scale, at short time delays the signal lineshapes are markedly sharper than those in the linear absorption spectrum. For both ligands, crosspeaks are resolved below the diagonal of the 2DES spectrum at the excitation energy of the X3 state and at the detection energies of the X2 and X1 states. The cross peaks indicate the transfer of population from the X3 state to the two lower exciton states via an ultrafast process.

**Figure 1:**
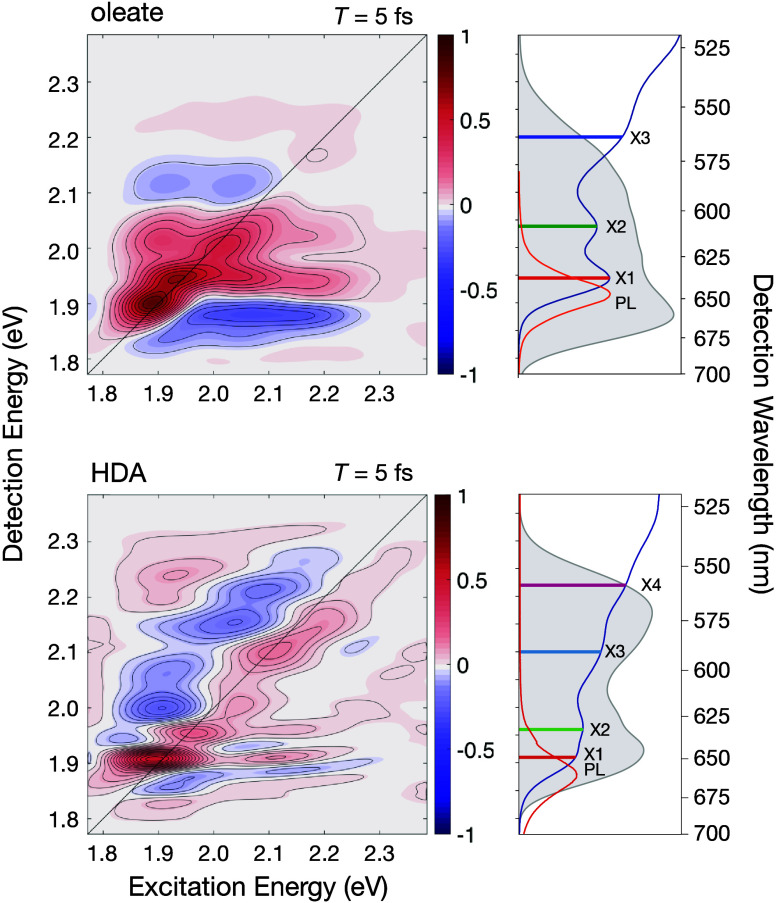
2DES spectra at delay *T* = 5 fs from CdSe QDs with oleate or hexadecylamine (HDA) surface-capping ligands, from the set of spectra discussed previously.^16,17^ The side panels to the right of the two 2DES spectra show the linear absorption oscillator strength (ε/ν, blue), photoluminescence (PL, orange), and laser spectra (gray-shaded); red, green, blue, and magenta bars mark the transition energies estimated from the absorption spectrum for the lowest exciton states: X1 (1S_3/2_–1S_e_), X2 (2S_3/2_–1S_e_), X3 (1P_3/2_–1P_e_), and X4 (2*S*_1/2_–1S_e_).^20^ The 2DES contours are shaded with respect to the indicated color bar relative to the full-scale signal amplitude of the data set for each ligand. The 2DES spectra have not been corrected for the intensity of the excitation and detection laser pulses.

An assignment of the electronic and vibrational processes underlying the time evolution of the 2DES spectra due to intraband internal conversion in the presence of the two ligands can be obtained from global models^21^ of the time evolution of the 2DES signal amplitude with state-specific preparation of each of the X1–X3 exciton states. The overall signal amplitude, *A*(*E*_det_, *T*) = *∑*_*n*_ *P*_*n*_(*T*)*S*_*n*_(*E*_det_), is determined in these models as the sum of the signals from a set of *n* spectrokinetic species in a kinetic model for a linear nonradiative decay pathway. The sum is determined as a linear combination of a set of basis spectra with respect to the detection energy, *S*_*n*_(*E*_det_), which are scaled by the populations *P*_*n*_ with respect to the probe delay *T*. The first species in the model is that produced instantaneously by the optical transition to the photoselected exciton state, and the last species is the final excited state populated prior to recovery to the original electronic ground state.

Figure 2 shows the basis spectra, *S*_*n*_(*E*_det_), which are termed evolution-associated difference spectra (EADS),^21^ for the optimized global model of the slice of the 2DES spectrum at the excitation energy of the X3 state. These spectra are interpreted as representing the third-order nonlinear optical spectra with respect to the detection energy axis in the 2DES spectrum due to each of the spectrokinetic species in the model. A minimally determined, approximate model for the response of the oleate-capped QDs out to *T* = 100 ps requires a model with five species, whereas that for the HDA-capped QDs requires only four species. The number of species required to obtain an adequate model was assessed by viewing the residual signal amplitude (signal – model) in a series of transients sampled at the detection energies of the X3 state near the diagonal and of the X1 and PL states.

**Figure 2:**
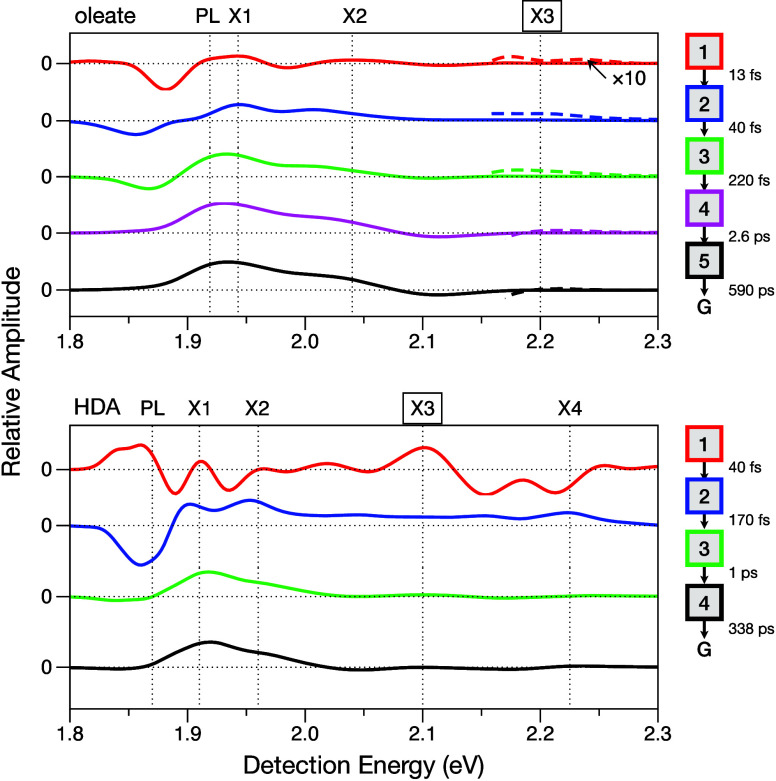
Evolution-associated difference spectra (EADS) from global models of the 2DES spectra from oleate- and HDA-capped CdSe QDs with excitation of the X3 transition. The spectra correspond to the spectrokinetic components of the global models shown in the legends to the right of the spectra. Component 1 corresponds to the instantaneously produced population prepared optically by the excitation pulses; the subsequent components are produced by nonradiative decay with the indicated time constants, with G standing for the final ground state. The detection energies for the photoluminescence (PL) and X1–X4 transitions are marked in each set with vertical dotted lines. Figures S1 and S2 show the EADS for the global models for excitations at the X1 and X2 transitions.

The electronic part of the hot-carrier cooling process shifts the positive-going signal due to stimulated emission (SE) pathways to lower detection energies, starting from the photoselected X3 state and ending near the band-edge X1 and PL energies. This ultrafast process principally accompanies the relaxation of population from the first to the second spectrokinetic species in the global models, with estimated time constants of 13 ± 2 fs and 40 ± 2 fs for the oleate- and HDA-capped QDs, respectively. Assignment of the SE contribution to the EADS is aided by inspection of the global models corresponding to excitation of the X2 and X1 states, which are shown in Figures S1 and S2 for the oleate- and HDA-capped CdSe QDs, respectively. As the population in the optically prepared state relaxes from X3, the SE signal amplitude increases at X2 and at the band-edge X1 state. Ground-state bleaching (GSB) signals persist during this process at least over the detection energy range of the X1–X3 states due to their sharing of the same electronic ground state. The positive GSB and SE signals are superimposed over the PL–X3 range with a negative signal due to a broad excited-state absorption (ESA) band, which arises here from electric-dipole-allowed transitions driven by the probe pulse’s field–matter interaction to a manifold of doubly excited states.^22,23^

The ultrafast red shift of the SE signal after optical preparation of the X3 state is followed by a slower spectral red-shifting and narrowing process that increases the signal amplitude at the detection energy of the band-edge X1 state. This accompanies relaxation from the second and third to the fourth spectrokinetic species in the global model for the oleate-capped QDs, with 40 ± 2 fs and 220 ± 5 fs time constants, respectively; a similar evolution of the EADS occurs during the conversion of the second to the third species in the model for the HDA-capped QDs with a 170 ± 5 fs time constant. This process can be attributed to vibrational cooling of the population in the X1 state, which is consistent with the observation that the net ESA part of the signal shifts to the blue at the same time as the net positive signal amplitude increases at X1 and lower detection energies due to SE. In both models, the EADS lineshapes for the subsequent spectrokinetic species are then essentially constant over the >100 ps time scale, which accompanies interband relaxation recovering the original electronic ground state. Because of the relatively sparse sampling of the probe delay *T* axis beyond 800 fs and 1000 fs for the oleate and HDA data sets, respectively, the picosecond and longer time constants in both of these models should be regarded only as rough estimates.

Inspection of the amplitude transients plotted in Figure 3 makes it clear that excited-state vibronic coherences involving the surface-capping ligands are rapidly damped mainly during the initial blue shift of the SE that marks the electronic part of the hot-carrier cooling process, which accompanies the decay of the population in the first kinetic compartment of the global models. As reported elsewhere in an analysis of coherences,^17^ the oleate-capped QDs exhibit a pair of relatively strong modulation components due to excited-state vibronic coherences at 145 cm^–1^ and 375 cm^–1^, which are assigned to a mixture of the LO phonon with a carboxylate mode and to a wagging vibration of the OCO moiety of the carboxylate and/or a bending vibration of the alkylcarboxylate CCO segment, respectively. An analogous pair of modulation components is observed in the HDA-capped QDs,^16^ with that at 450 cm^–1^ likely corresponding to a bending deformation of the alkylamine moiety of the ligand.^18^ The <50 fs damping times exhibited by these modulated signals are somewhat longer than the time constants for the electronic processes in the global models. At the diagonal (X3, X3) coordinate of the 2DES spectrum, nearly a full recurrence of the signal amplitude is observed in the HDA-capped QDs over the *T* < 50 fs range, whereas the oleate-capped QDs exhibit a much weaker modulation only over the rise of the transient. The initial beating signal detected in the oleate-capped QDs at the (X3, PL) coordinate subsides in ∼100 fs, whereas that for the HDA-capped QDs is damped out at ∼150 fs. As explained in the following, these observations indicate that the vibrational motions of wavepackets along the trajectory from the Franck–Condon geometry in the X3 state to the lower exciton states are damped more rapidly in the oleate-capped QDs because of a more rapid passage through a series of CIs.

**Figure 3:**
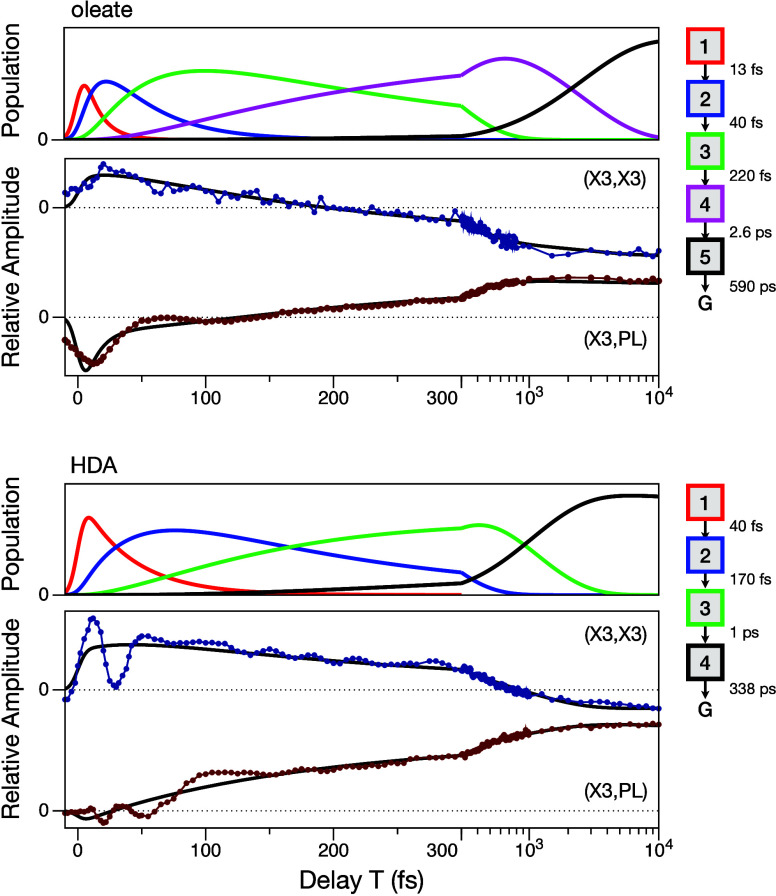
Amplitude transients observed at the (X3, X3) and (X3, PL) coordinates of the 2DES spectra from the oleate- and HDA-capped CdSe QDs. The data points are superimposed with the fitted signal amplitude of the global model as a function of the delay *T* (black curves). The populations correspond to the spectrokinetic species and time constants in the global model, as indicated by the legend to the right.

The cartoon shown in Figure 4 describes the nonlinear optical response of the CdSe QDs following excitation to the X3 state in terms of coherent wavepacket motions on the ground and excited exciton potential energy surfaces with respect to the vibrational coordinates of the QD–ligand complex. Stimulated Raman coherences, resulting in wavepackets transferred to the electronic ground state during the instrument-response function of the 2DES experiment, provide a probe of the coordinates that are instantaneously displaced upon optical excitation from the geometry of the equilibrium ground state.^24^ Ligand-specific vibrations have been observed previously in the resonance Raman spectra of QDs,^25^ and calculations of resonance Raman intensities have considered the bonding character of the ligands to the surface of the QD.^26,27^ A progression of stimulated Raman coherences in the oleate-capped QDs establishes that the LO phonon of the QD core is mixed with alkylcarboxylate modes.^17^ After the initial displacement, the excited-state wavepackets are directed through a series of CIs by the potential energy gradients between them with respect to the branching (or *tuning* and *coupling*) modes,^28^ which vary the energy gap and the strength of interaction between two diabatic potential surfaces.^29–31^ The CIs are actually *seams* of dimensionality *N* – 2, where two surfaces intersect as the other *N* vibrational coordinates vary.^30,32^

**Figure 4:**
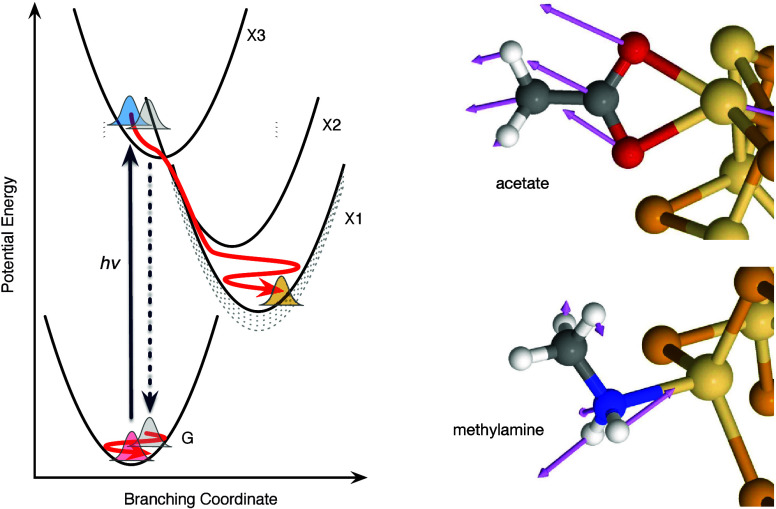
(left) Coherent nonadiabatic relaxation mechanism following optical preparation of the X3 exciton state in the oleate-capped CdSe QDs. Excited-state wavepackets travel from the Franck–Condon structure (blue wavepacket) to the band-edge X1 state (orange wavepacket) by passing through a sequence of CIs of the X3 and X2 and X2 and X1 potential energy surfaces. Ground-state wavepacket motions arise from stimulated Raman pathways due to an instantaneous displacement (gray wavepackets) from the Franck–Condon structure. (right) Structures of acetate and methylamine ligands coordinated to surface Cd^2+^ ions from the ground-state structures of Cd_33_Se_33_ nanocrystals, as discussed previously.^18,19^ Normal mode displacement vectors are shown for the 300 cm^–1^ mode of the acetate ligand and for the 474 cm^–1^ mode of the methylamine ligand.

The excited-state trajectory depicted in Figure 4 attempts to describe the situation evident in the oleate-capped QDs, where the excited-state wavepackets appear to cross almost instantaneously to a lower exciton state’s potential surface, either that of X2 on the way to X1 or directly to X1. Because the coherence apparently decays following the passage of the wavepacket through the CI(s), the rate of damping of the excited-state coherences is connected to the rate of passage from the initial exciton state to a lower-energy product exciton state. This kind of behavior of the coherence during fast reactions has been noted previously.^33–35^ A recent theoretical discussion^36^ suggests that the dephasing arises from a *quantum quench*,^37^ effectively a rapid change in the forces acting on the system that would accompany the change in the system’s Hamiltonian upon crossing through the CI. In the HDA-capped QDs, the diagonal (X3, X3) transient shows that the crossing from the X3 state’s potential energy surface is slower than in the oleate-capped QDs, allowing perhaps a full recurrence of the wavepacket back to the Franck–Condon geometry before it passes through a CI to a lower-energy state. The observations here suggest that the dephasing time constant is generally somewhat longer than that of the nonradiative decay process, given that we can detect residual oscillations due to the rapidly damped modes in the product state. This emphasizes that it is not the change in populations going from the reactant to the product state^36^ but rather the change in the electronic structure that is sensed by the dephasing of the branching modes near the CIs. The relative phase of the transient detected at PL compared to that detected at X3 is accordingly a measure of the transit time for the wavepacket motion through the CIs. Overall, the amplitudes of these coherences would be expected to depend on the trajectory taken through the CIs,^38^ as determined by the potential energy gradient through them but also by the strength of the nonadiabatic coupling.

The subsequent vibrational cooling process we observe over the ∼500 fs time scale can be assigned to the energy relaxation that follows the arrival of wavepackets well above the X1 minimum. The 200 fs time constant estimated here from the global models is consistent with IVR in large organic molecules,^39–41^ which is reasonable given that here it would involve the transfer of vibrational energy between the active and spectator modes local to the ligands, but similar time scales have also been observed in coordination complexes.^42–44^ The spectator modes would be expected to retain vibrational coherence upon reaching X1 because their displacements do not cause passage through the CIs between the exciton states. Damping of these modes in X1 can be attributed mainly to IVR rather than to pure vibrational dephasing, which would be expected to occur on the picosecond time scale.

These findings are significant because it is now evident that only the initial electronic part of the hot-carrier cooling mechanism is specific to a particular ligand, which suggests an important role of coherent ligand vibrations with charge-transfer character in the mechanism of photoinduced charge transfer or triplet–triplet excitation energy transfer to surface-bound acceptors. The rapidly damped excited-state vibrations of the ligands may function as tuning modes in a coherent nonadiabatic mechanism because their displacements will cause gradients of the exciton potential energy surfaces near the CIs due to their bonding interactions on the surface of the QDs, particularly due to mixing with the hole part of the exciton wave function in the valence band.^27,45–47^ Steeper potential energy gradients and higher rates of electronic relaxation might be expected because the oleate’s carboxylate is a somewhat harder, negatively charged ligand with π-donating character in comparison to the neutral alkylamine ligand’s σ-donating character. Conversion of the electronic energy of the X3 state accompanies a few ligand vibrational periods at the most, so it is possible that intermediary states with core-to-ligand charge-separated character will be produced with retention of vibrational coherence prior to a full transfer of electrons to surface-bound acceptors on the picosecond and longer time scale. This concept anticipates that the yield of photoinduced charge transfer and of triplet–triplet excitation energy transfer will be controlled by the excited-state trajectory taken from the photoexcited X3 state through the CIs that lead to charge-separated intermediates, with mixing of the QD core and surface acceptor orbitals modulated by the mid-frequency vibrations of the latter.

## Methods

CdSe QDs with HDA and oleate ligands were prepared and characterized by the Van Patten and Zhang laboratories at Middle Tennessee State University, as discussed in the previous publications on the 2DES data sets analyzed in this contribution.^16,17^

Broadband two- and three-dimensional electronic spectra (2DES and 3DES) data sets of the HDA- and oleate-capped QDs were reported previously.^16,17^ Both data sets were recorded with ∼7 fs pulses at ambient temperature (293 K) with a two-beam, pump–probe spectrometer employing adaptive pulse shaping^48^ and phase-sensitive detection.^49^ Global modeling of the 2DES spectra for the present paper was performed with the CarpetView program (Light Conversion). For the oleate data set, the probe delay *T* axis was sampled at 2.5 fs steps over the −20 to 20 fs range and then with 5 fs steps up to 400 fs and 10 fs steps up to 800 fs; 500 fs and 1 ps steps were used over the 1–2 ps and 2–10 ps ranges, respectively. The *T* axis was sampled in the HDA data set with 2.5 fs steps over the −25 –50 fs range and then with 5 fs steps up to 500 fs and 10 fs steps up to 1000 fs; 250 fs, 500 fs, and 1 ps steps were then used over the 1–2, 2–5, and 5–10 ps ranges, respectively. Confidence intervals for the time constants in the spectrokinetic models were estimated for the *T* < 1 ps components by performing separate global models for the individual *T* delay scans in the data sets. Because the sampling of the *T* axis is relatively sparse, we only provide rough estimates for the time constants for the picosecond and slower components, including that for ground-state recovery.

## Supplementary Material



## Data Availability

The data sets and the MATLAB, Julia, and Python code used to analyze the results reported in this paper are available in an archive with DOI: 10.5281/zenodo.17924931.

## Notes

EADS for global models of the 2DES spectra from oleate- and HDA-capped CdSe QDs with excitation of the X1, X2, and X3 exciton states (PDF)

## §

Applied Materials, Inc., 3050 Bowers Avenue, Santa Clara, CA 95054, United States

## ∥

The Dow Chemical Company, 1897 Building, E75, 633 Washington Street, Midland, MI 48674, United States
