# Supplementary material for: Ligand Control of Ultrafast Hot-Carrier Cooling in CdSe Quantum Dots by a Coherent Nonadiabatic Mechanism
Source: J Phys Chem Lett. 2026 Jan 12;17(4):1055–61. doi: 10.1021/acs.jpclett.5c03429 (PMC12862819; doi:10.1021/acs.jpclett.5c03429)
Supplement: Supplementary file 1 [file jz5c03429_si_001.pdf]

Supplemental Information

Ligand control of ultrafast hot-carrier cooling in CdSe quantum dots by a coherent nonadiabatic mechanism

Shanu A. Shameem[1,a], Nila Mohan T. M.[1,a,b], Ryan W. Tilluck[1,c], Caitlin V. Hetherington[2], Benjamin G. Levine[2], and Warren F. Beck[1,*]

[1]Department of Chemistry, Michigan State University
578 S. Shaw Lane, East Lansing, Michigan 48824 U.S.A.

[2]Institute for Advanced Computational Science and Department of Chemistry, Stony Brook University, Stony Brook, New York 11794 U.S.A.

[a]Equal contributions.

[b]Current address: Applied Materials, Inc., 3050 Bowers Avenue, Santa Clara, CA 95054, U.S.A.

[c]Current address: The Dow Chemical Company, 1897 Building, E75, 633 Washington Street, Midland, MI 48674, U.S.A.

[*]Corresponding author. Email: beckw@msu.edu

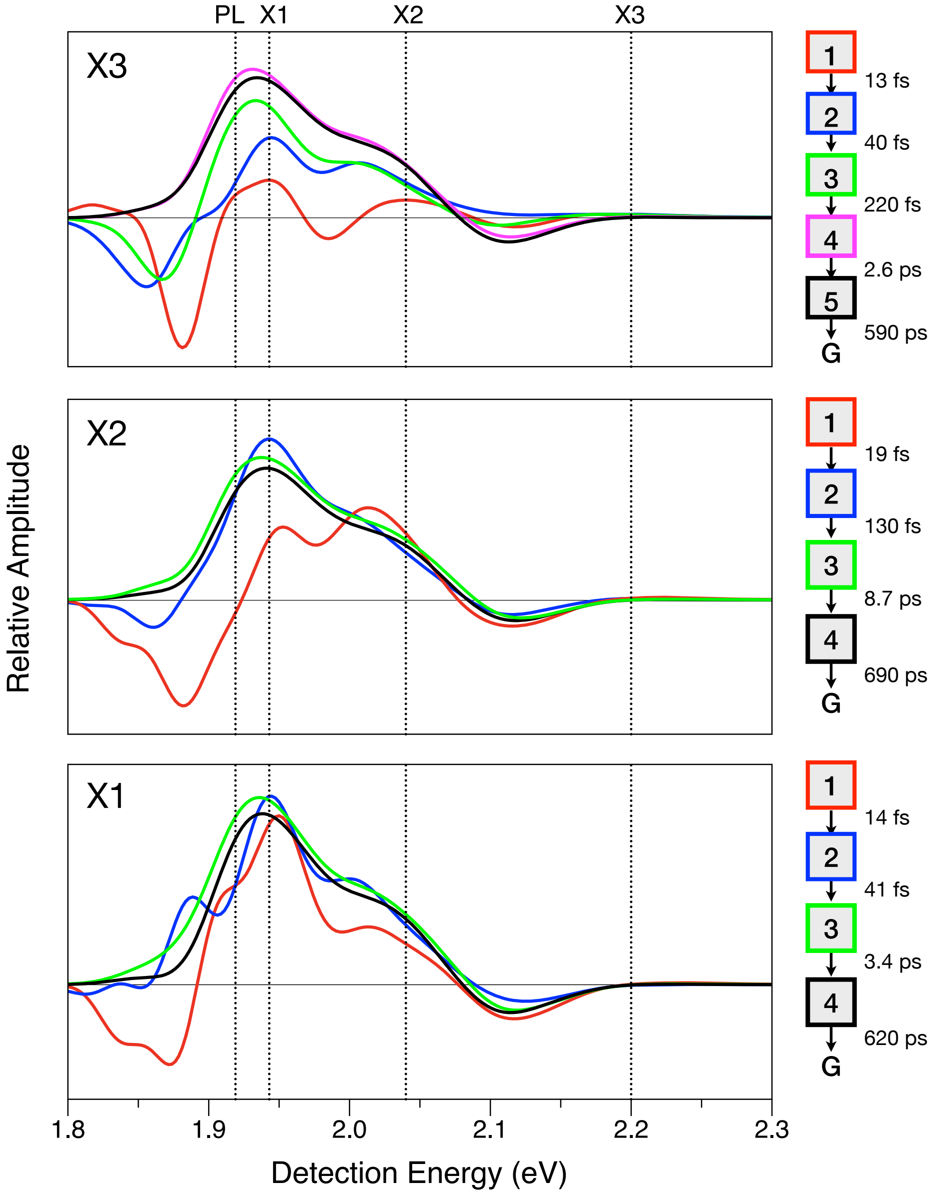

Fig. 1. Evolution-associated difference spectra (EADS) from global models of the 2DES spectra from oleate-capped CdSe QDs with the responses selected for excitation at the X1, X2, or X3 transition. The spectra correspond to the spectrokinetic components of the global models shown in the legends to the right of the spectra. Component 1 corresponds to the instantaneously produced population prepared by the excitation pulses, whereas components 2–4 and –5 are the subsequent components produced by nonradiative decay, with G standing for the final ground state. The detection energies for the photoluminescence (PL) and X1–X4 transitions are marked in each set with vertical dotted lines.

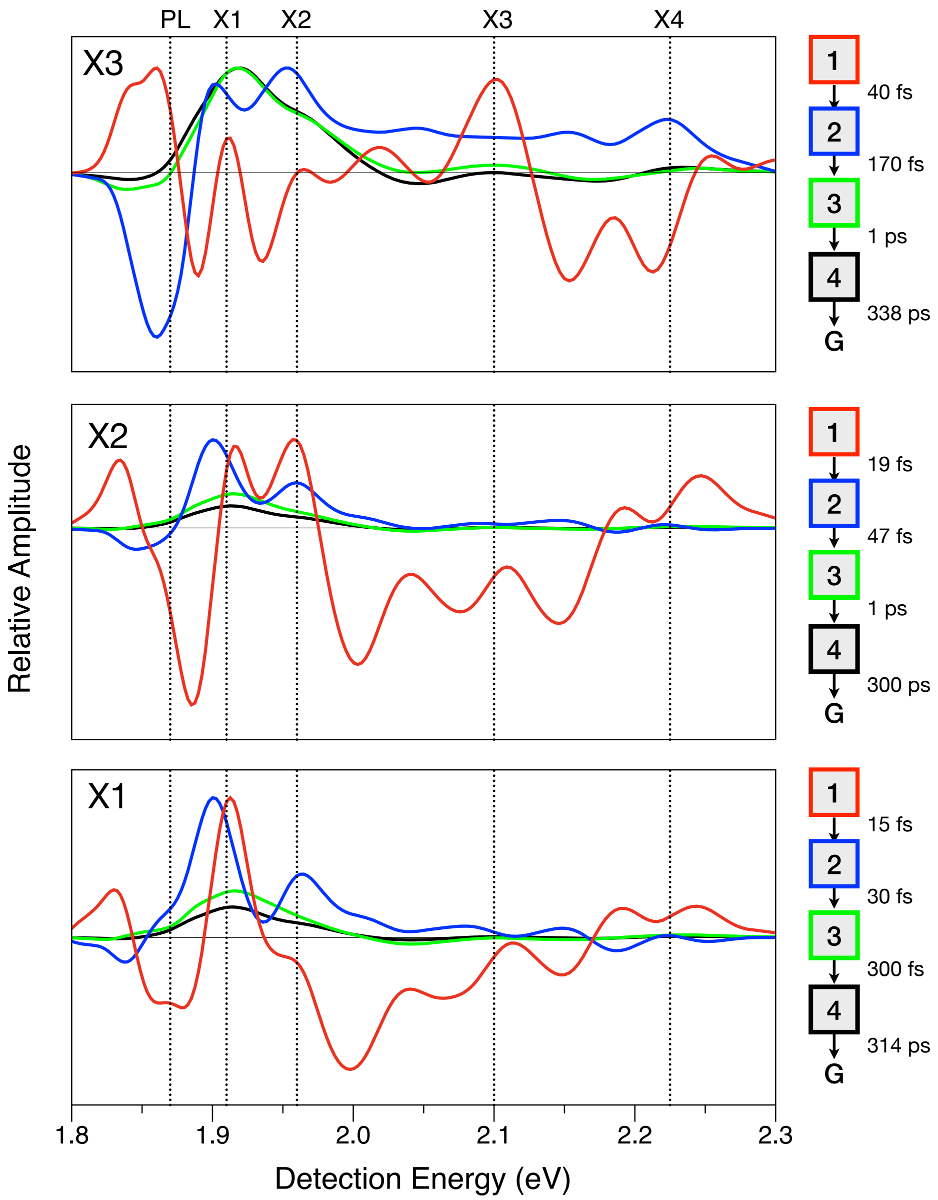

Fig. 2. Evolution-associated difference spectra (EADS) from global models of the 2DES spectra from HDA-capped CdSe QDs with the responses selected for excitation at the X1, X2, or X3 transition. The spectra correspond to the spectrokinetic components of the global models shown in the legends to the right of the spectra. Component 1 corresponds to the instantaneously produced population prepared by the excitation pulses, whereas components 2–4 and –5 are the subsequent components produced by nonradiative decay, with G standing for the final ground state. The detection energies for the photoluminescence (PL) and X1–X4 transitions are marked in each set with vertical dotted lines.
